# Aberrant WNT/β-catenin signaling in parathyroid carcinoma

**DOI:** 10.1186/1476-4598-9-294

**Published:** 2010-11-15

**Authors:** Jessica Svedlund, Maria Aurén, Magnus Sundström, Henning Dralle, Göran Åkerström, Peyman Björklund, Gunnar Westin

**Affiliations:** 1 Department of Surgical Sciences, Endocrine Unit, Uppsala University, SE-751 85 Uppsala, Sweden; 2 Department of Genetics and Pathology, Uppsala University, SE-751 85 Uppsala, Sweden; 3 Department of General Surgery, Martin-Luther-University of Halle-Wittenberg, 06097 Halle/Saale, Germany

## Abstract

**Background:**

Parathyroid carcinoma (PC) is a very rare malignancy with a high tendency to recur locally, and recurrent disease is difficult to eradicate. In most western European countries and United States, these malignant neoplasms cause less than 1% of the cases with primary hyperparathyroidism, whereas incidence as high as 5% have been reported from Italy, Japan, and India. The molecular etiology of PC is poorly understood.

**Results:**

The APC (adenomatous polyposis coli) tumor suppressor gene was inactivated by DNA methylation in five analyzed PCs, as determined by RT-PCR, Western blotting, and quantitative bisulfite pyrosequencing analyses. This was accompanied by accumulation of stabilized active nonphosphorylated β-catenin, strongly suggesting aberrant activation of the WNT/β-catenin signaling pathway in these tumors. Treatment of a primary PC cell culture with the DNA hypomethylating agent 5-aza-2'-deoxycytidine (decitabine, Dacogen(r)) induced APC expression, reduced active nonphosphorylated β-catenin, inhibited cell growth, and caused apoptosis.

**Conclusion:**

Aberrant WNT/β-catenin signaling by lost expression and DNA methylation of APC, and accumulation of active nonphosphorylated β-catenin was observed in the analyzed PCs. We suggest that adjuvant epigenetic therapy should be considered as an additional option in the treatment of patients with recurrent or metastatic parathyroid carcinoma.

## Background

Parathyroid carcinoma (PC) is a rare cause of primary hyperparathyroidism with characteristic marked hypercalcemia and highly elevated parathyroid hormone levels. In most western European countries and United States, these malignant neoplasms cause less then 1% of the cases, whereas incidence as high as 5% have been reported from Italy, Japan, and India [1-4]. The incidence in the United States has more than doubled since 1988 [5]. PCs are slow-growing with a high tendency to recur locally (30%-50%), and recurrent disease is difficult to eradicate [6-8]. An overall 5-year survival rate of 86% and a 10-year survival rate of 49% have been observed in a report that described 286 cases of PC in the United States [9].

Loss of heterozygocity analyses have shown a higher fractional allelic loss in PCs than in parathyroid adenomas, where frequently deleted regions included the PTEN, RB, HRAS, p53, MEN1, and HRPT2 genes [10,11]. Somatic MEN1 mutations were recently described in 3 of 23 (13%) PCs, suggesting a role in the development of these tumors [12]. Overexpression of the cyclin D1 oncogene (CCND1) has been observed in 10 of 11 (91%) PC specimens [13]. Carriers of the hyperparathyroidism-jaw tumor (HPT-JT) syndrome show a higher risk of developing PC; 10% to 15% of affected individuals present with malignant parathyroid tumors [14]. Somatic mutations of HRPT2 (parafibromin), the gene responsible for HPT-JT syndrome [14], occur in PC with an estimated odds ratio of 45.2 (95% CI: 12.8-160.0%) as calculated from a compilation of six published studies. The HRPT2 mutation frequency in sporadic parathyroid adenoma was calculated to 1.8% [15].

Negative immunostaining for parafibromin has been suggested as a means to diagnose PC, if the rare commonly cystic adenomas of the HPT-JT syndrome are excluded [16,17]. Recently, it was suggested that this may have some limited validity and cannot replace HRPT2 mutation analysis [18]. Positive immunostaining for PGP9.5 (UCHL1) as well as absent staining for the adenomatous polyposis coli (APC) tumor suppressor have been suggested recently as additional markers for parathyroid malignancy [19,20].

Inactivation of APC by mutation with subsequent stabilization of β-catenin has been strongly implicated in the cause of approximately 80% of colorectal cancers [21-24]. APC function can also be impaired by reduced expression through hypermethylation of the APC 1A promoter, a phenomenon observed in several malignances [25-29].

In the present study, we show that absent or markedly reduced expression of APC in five analyzed PCs is caused by hypermethylation of the APC promoter 1A. We also show that this was accompanied by accumulation of stabilized active nonphosphorylated β-catenin, strongly suggesting aberrant activation of the WNT/β-catenin signaling pathway in these tumors. Treatment of a primary PC cell culture with the DNA hypomethylating agent 5-aza-2'-deoxycytidine induced APC expression, reduced active nonphosphorylated β-catenin, inhibited cell growth, and caused apoptosis. 5-aza-2'-deoxycytidine (decitabine; Dacogen; MGI Pharma, Bloomington, MN) was recently approved by the United States Food and Drug Administration for the treatment of myelodysplastic syndrome [30,31]. We suggest that epigenetic therapy should be considered as an additional option in the treatment of patients with recurrent or metastatic parathyroid carcinoma.

## Methods

### Tissue specimens

Tissue specimens were acquired from patients diagnosed and operated on in the clinical routine at Uppsala University Hospital and Martin-Luther-University of Halle-Wittenberg. The tissue was intraoperatively snap frozen. Normal parathyroid tissue was obtained from glands inadvertently removed in conjunction with thyroid surgery where autotransplantation was not required (n = 1) or as normal parathyroid gland biopsies in patients subjected to parathyroidectomy (n = 7). The diagnosis of parathyroid carcinoma (n = 5) was unequivocal due to occurrence of distant metastases at diagnosis or follow-up. Clinical characteristics of four PC patients have been published [32]. Written informed consent was obtained from the patients and approval was obtained from the local ethical committee, Uppsala.

### DNA and RNA preparation, RT-PCR

DNA was prepared from cryosections using QIAamp(r) DNA Mini Kit (Qiagen) and total RNA using TriZol Reagent (Gibco BRL, Life Technologies Inc.), according to the manufacturer's instructions. DNA-free RNA was prepared using the Nucleospin RNA II kit (Macherey-Nagel GmbH & Co. KG). Reverse transcription of RNA was performed with random hexamer primers using the First-Strand cDNA Synthesis kit (GE Healthcare) according to the manufacturer's instructions. RT-PCR of APC was performed using the forward primer 5'-gccagctcctgttgaacatcagat (exon 11) and the reverse primer 5-gcccatacatttcacagtccactt (exon 12). The PCR resulted in a 150 base pair fragment. Primers for GAPDH were as described previously [32].

### Quantitative RT-PCR

Quantitative real-time PCR was performed on StepOnePlus™ Real-Time PCR systems (Applied Biosystems, Foster City, CA) using assays for APC (Hs 01568270_m1) and GAPDH (Hs99999905_m1). Each cDNA sample was analyzed in triplicate. Standard curves were established by amplifying a purified PCR fragment covering the sites for probes and primers.

### Immunohistochemistry

Frozen tissue sections were stained as described [33] using an anti-APC mouse monoclonal antibody with an epitope mapping at the N-terminus (Santa Cruz Biotechnology, INC., Santa Cruz, CA; catalog no. sc-9998).

### DNA sequencing

All APC coding exons 2-15 with intron/exon junctions were directly sequenced on the 3130*xl *Genetic Analyzer using the Big Dye(r) terminator v1.1 cycle sequencing kit (Applied Biosystems, Foster City, CA). PCR primers for exon 10 and exon 14 [34], and the additional ones [35] were as published. The known C/T (exon 12; position 14577867) and G/A polymorphisms (exon 14; position 14579574) were found in three and five of the analyzed PCs, respectively. No mutations were observed.

### Bisulfite pyrosequencing

DNA was converted using the EpiTect Bisulfite Kit and PCR amplified using the HotStarTaq *Plus *Master Mix Kit (Qiagen). The APC assay used is available at the PyroMark Assay Database (Qiagen). Pyrosequencing [36-38] was done with the PyroMark™ Q24 system (Qiagen). All samples were analyzed in triplicates (Additional file 1: Table S1).

### Western blotting analysis

Western blotting analyses were done on extracts prepared in Cytobuster Protein Extract Reagent (Novagen Inc., Madison, Wisconsin, USA) or RIPA buffer supplemented with Complete protease inhibitor cocktail (Roche Diagnostics GmbH, Mannheim, Germany). Anti-β-catenin goat polyclonal antibody (Santa Cruz Biotechnology Inc., Santa Cruz, USA, # sc-1496), anti-active-β-catenin [39] (Upstate, Lake Placid, USA, # 05-665), anti-APC rabbit polyclonal antibody (Santa Cruz Biotechnology Inc., Santa Cruz, USA, # sc-896), and anti-β-tubulin polyclonal antibody (Santa Cruz Biotechnology Inc.) were used. After incubation with the appropriate secondary antibodies, bands were visualized using the enhanced chemiluminescence system (GE Healthcare Europe GmbH, Uppsala, Sweden).

### Cell culture

Parathyroid carcinoma cells (PC1) were prepared directly after operation according to published procedures [40]. Collagenase digestion was for 1 hour. Cells (2×10^5^) were distributed onto 35-mm dishes in DMEM/10% fetal bovine serum (Sigma) and incubated in triplicates with 5 μM 5-aza-2'-deoxycytidine (Sigma-Aldrich). Fresh 5-aza-2'-deoxycytidine was added every 24 hrs. Cells were harvested after 24 hrs and 48 hrs for RNA and protein extraction. Cell viability was measured on PC1 cells distributed in triplicates (2×10^4^) onto 96 well plates using the cell proliferation reagent WST-1 (Roche Diagnostics GmbH, Mannheim, Germany). Quantification of cytoplasmic histone-associated-DNA-fragments (mono- and oligonucleosomes) was done using the Cell Death Detection ELISA PLUS kit (Roche Diagnostics). Camptothecin at 0.1 μg/ml was used as positive control of apoptosis.

### Statistical analysis

Unpaired *t *test was used. Values are presented as arithmetical mean ± SEM. A p value of < 0.05 was considered significant.

## Results and Discussion

### APC promoter hypermethylation in parathyroid carcinoma

**Figure 1:**
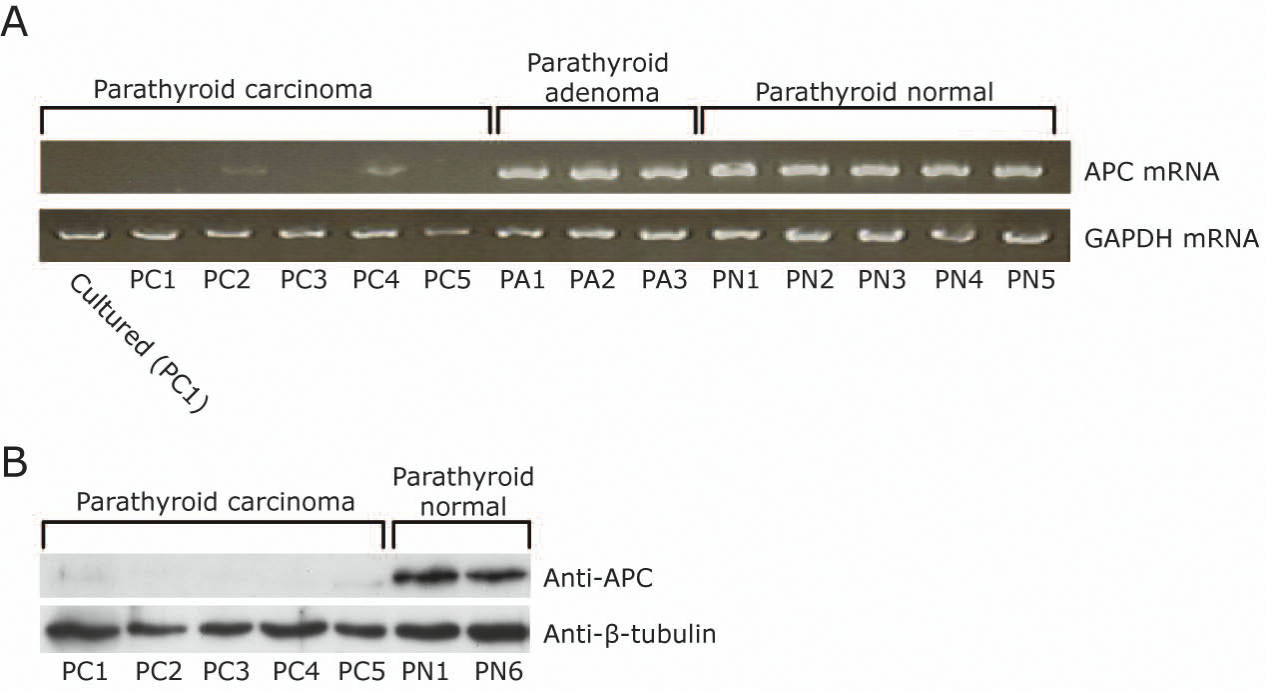
**Expression of APC mRNA and protein**. A, RT-PCR analysis of parathyroid carcinomas (PC1-5), parathyroid adenomas (PA1-3), and normal parathyroid tissue specimens (PN1-5). B, Western blotting analysis of APC.

**Figure 2:**
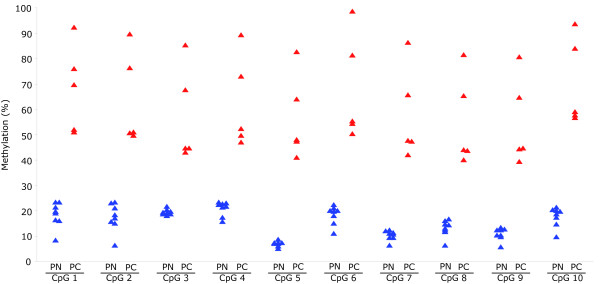
**DNA methylation analysis by quantitative bisulfite pyrosequencing**. Parathyroid carcinomas (in red; n = 5) and normal parathyroid tissue specimens (in blue; n = 8). Methylation levels were determined for ten CpGs of the APC promoter 1A, in triplicates. See Additional file 1, Table S1 for all measurements.

**Figure 3:**
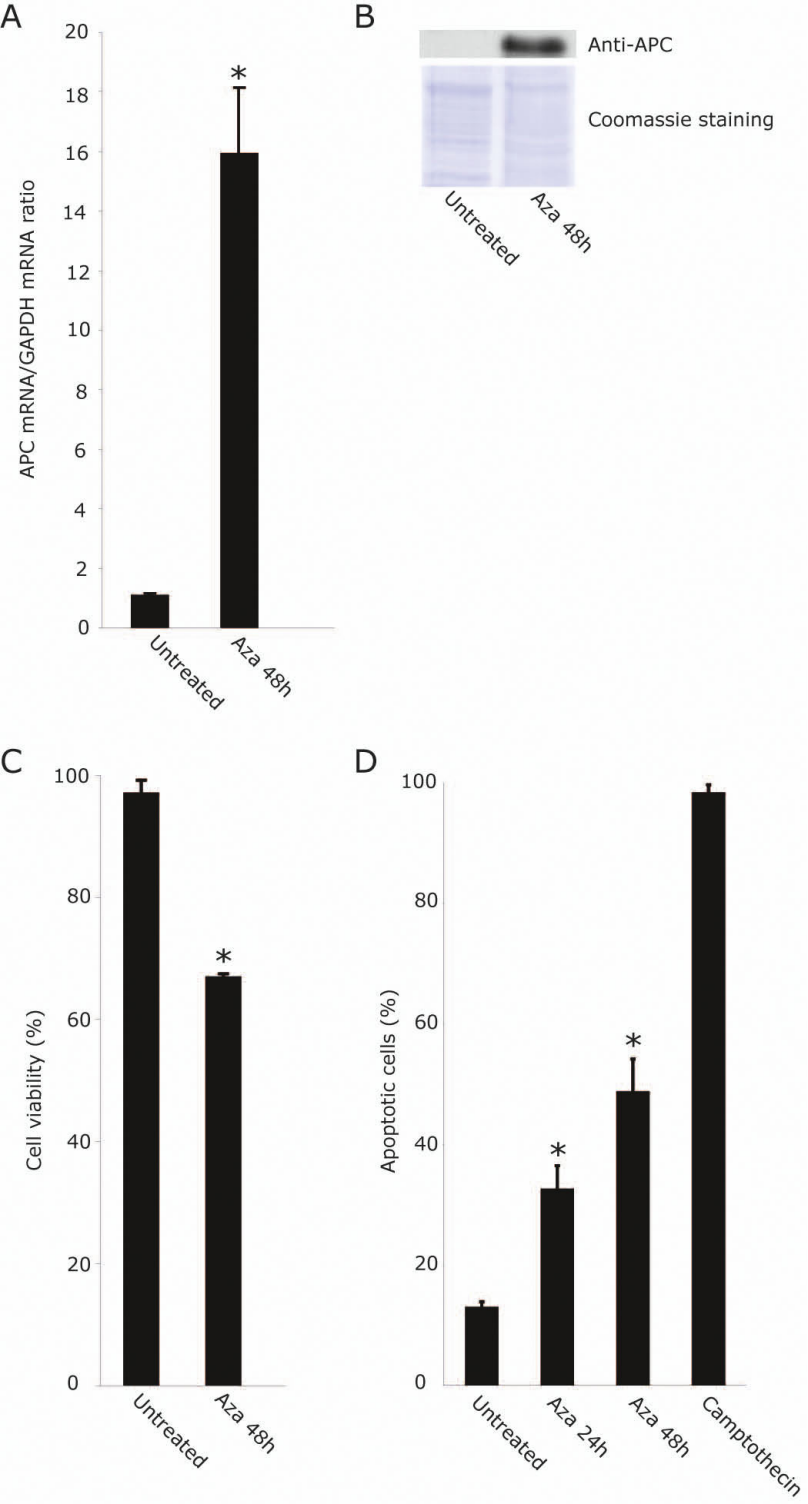
**Effects of 5 μM 5-aza-2'-deoxycytidine (Aza) on *in vitro *cultured primary parathyroid carcinoma cells (PC1)**. A, APC/GAPDH mRNA expression ratio as determined by real-time quantitative RT-PCR analysis. B, Western blotting analysis of APC. Coomassie blue-stained filter is shown as loading control. C, cell viability. D, apoptosis. Camptothecin at 0.1 μg/ml was used as positive control.

In order to determine whether expression of APC was perturbed in the five PC specimens, RT-PCR analysis was performed. The results showed that APC mRNA expression was undetectable to very low in the PCs when compared to parathyroid adenomas or normal parathyroid tissue specimens (Figure 1A). In agreement, APC protein expression was undetectable in the five PCs as revealed by Western blotting analysis (Figure 1B) and immunohistochemistry (data not shown). Gene inactivation or reduced expression could be caused by various mechanisms, including promoter hypermethylation. We used bisulfite pyrosequencing to quantitatively determine the methylation level for ten CpGs of the APC promoter 1A (Figure 2; Additional file 1, Table S1). The mean methylation levels (%) of all CpGs were significantly higher in the five PCs (48%-88%) than in the eight normal parathyroid tissue specimens (10%-18%). It is conceivable that the increased methylation level of the APC promoter caused the prominent reduction in gene expression. This was further supported by *in vitro *cell culturing experiments (Figure 3). Treatment of cultured primary cells from parathyroid carcinoma no. 1 (PC1) with the DNA methylation inhibitor 5-aza-2'-deoxycytidine (decitabine, Dacogen(r)) resulted in apparent induction of APC mRNA and protein expression, and as expected [41] also in reduced cell viability and apoptosis (Figure 3A-D). Thus, a mean APC methylation level of 48% (PC1; Additional file 1, Table S1) seemed sufficient for "gene inactivation". We note that this is in agreement with a recent report on a number of hypermethylated genes in colon cancer where methylation levels in excess of 40% were found to be incompatible with transcription [42]. We also performed DNA sequencing analysis of all APC exons and intron/exon splice junctions in the five PCs, with no detected mutations (data not shown).

### Accumulation of active nonphosphorylated β-catenin in parathyroid carcinoma

**Figure 4:**
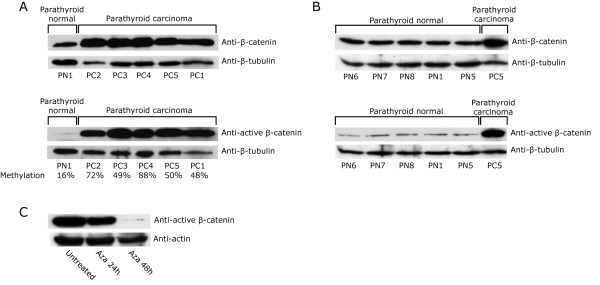
**Western blotting analysis of total β-catenin (Anti-β-catenin) and active nonphosphorylated β-catenin (Anti-active β-catenin)**. A, the five parathyroid carcinomas. Mean methylation levels (Additional file 1, Table S1) of the APC promoter 1A are also shown. B, normal parathyroid tissue specimens (n = 5). C, active β-catenin level in cultured primary PC1 cells treated with 5-aza-2'-deoxycytidine for the indicated time points.

Mutation or vanished expression of APC may lead to aberrant activation of the WNT/β-catenin signaling pathway, through the accumulation of stabilized active nonphosphorylated β-catenin [21-24]. Western blotting analyses with antibodies to total β-catenin or to active nonphosphorylated β-catenin [39] showed almost similar relative protein levels in the five PCs (Figure 4A; B). On the contrary, in normal parathyroid tissue specimens (Figure 4B) the expression level of active nonphosphorylated β-catenin was lower than the total β-catenin level, and much lower than in the parathyroid carcinomas. These results strongly suggest the presence of accumulated active nonphosphorylated β-catenin in the analyzed PCs. Among its many functions APC controls the level of β-catenin [21-24]. We reasoned that if the strongly reduced expression of APC (Figure 1A-B) caused accumulation of active β-catenin, then re-expression of APC in cultured primary PC1 cells by treatment with the general demethylating agent 5-aza-2'-deoxycytidine (Figure 3A-B) should lead to a reduced active β-catenin level. A reduced active β-catenin level was observed (Figure 4C). This result is consistent with the normal role for APC in regulating the β-catenin level.

For all five analyzed PCs, our results strongly suggest inactivation of APC by DNA methylation and consequential aberrant WNT/β-catenin signaling by the accumulation of active nonphosphorylated β-catenin. Treatment *in vitro *with the hypomethylating agent 5-aza-2'-deoxycytidine inhibited PC cell growth and induced apoptosis, raising hopes for adjuvant therapy. A recent study demonstrated negative immunohistochemical staining for APC in nine of twelve PCs [20], supporting a frequent inactivation of APC in PCs.

The only treatment of PC with higher rate of cure is diagnosis before or during operation followed by complete removal of the tumor. Treatments of recurrent or metastatic disease have been difficult to evaluate due to the uncommonness of the tumor entity, but in one study adjuvant radiotherapy was shown to improve survival [43]. 5-aza-2'-deoxycytidine (decitabine; Dacogen; MGI Pharma, Bloomington, MN) was recently approved by the United States Food and Drug Administration for the treatment of myelodysplastic syndrome [30,31]. 5-aza-2'-deoxycytidine has been shown to suppress tumorigenecity of xenografted cells from solid tumors [41,44-46]. Although its efficacy in solid tumors remains to be fully evaluated [31,47], we suggest that Dacogen(r) should be taken under consideration for additional treatment of recurrent or metastatic PC, a life threatening condition which has been notoriously difficult to eradicate.

## Conclusions

The WNT/β-catenin signaling pathway was aberrantly activated in the analyzed parathyroid carcinomas, due to lost expression of APC likely caused by promoter DNA methylation. APC was reactivated *in vitro *by 5-aza-2'-deoxycytidine and adjuvant epigenetic therapy (decitabine; Dacogen; MGI Pharma, Bloomington, MN) should be considered as an additional option in the treatment of patients with recurrent or metastatic parathyroid carcinoma.

## Competing interests

The authors declare that they have no competing interests.

## Authors' contributions

JS performed experiments, analyzed data, performed statistical analysis, and helped to draft the manuscript. MA performed experiments, MS analyzed data and helped to draft the manuscript. HD contributed with material and helped to draft the manuscript. GÅ helped to draft the manuscript. PB performed experiments, analyzed data, performed statistical analysis, and helped to draft the manuscript. GW conceived of the study, participated in its design and coordination and drafted the manuscript. All authors read and approved the final manuscript.

## Supplementary Material

Additional file 1 **Table S1 - Quantitative bisulfite pyrosequencing of 10 CpGs in the APC promoter 1A**. Normal parathyroid tissue specimens (PN, n = 8) and parathyroid carcinoma (PC, n = 5) were analyzed in triplicate. Click here for file

## References

[B1] Obara T Fujimoto Y Diagnosis and treatment of patients with parathyroid carcinoma: an update and review World J Surg 1991 15 738 744 10.1007/BF01665308 1767540

[B2] Favia G Lumachi F Polistina F D'Amico DF Parathyroid carcinoma: sixteen new cases and suggestions for correct management World J Surg 1998 22 1225 1230 10.1007/s002689900549 9841748

[B3] Shane E Clinical review 122: Parathyroid carcinoma J Clin Endocrinol Metab 2001 86 485 493 10.1210/jc.86.2.485 11157996

[B4] Muthukrishnan J Jha S Modi KD Jha R Kumar J Verma A Harikumar KV Patro K Srinivas B Kumaresan K Ramasubba R Symptomatic primary hyperparathyroidism: a retrospective analysis of fifty one cases from a single centre J Assoc Physicians India 2008 56 503 507 18846900

[B5] Lee PK Jarosek SL Virnig BA Evasovich M Tuttle TM Trends in the incidence and treatment of parathyroid cancer in the United States Cancer 2007 109 1736 1741 10.1002/cncr.22599 17372919

[B6] Schantz A Castleman B Parathyroid carcinoma. A study of 70 cases Cancer 1973 31 600 605 10.1002/1097-0142(197303)31:3<600::AID-CNCR2820310316>3.0.CO;2-0 4693587

[B7] Sandelin K Auer G Bondeson L Grimelius L Farnebo LO Prognostic factors in parathyroid cancer: a review of 95 cases World J Surg 1992 16 724 731 10.1007/BF02067369 1413841

[B8] Iacobone M Ruffolo C Lumachi F Favia G Results of iterative surgery for Persistent and recurrent parathyroid carcinoma Langenbecks Arch Surg 2005 390 385 390 10.1007/s00423-005-0555-6 15933877

[B9] Hundahl SA Fleming ID Fremgen AM Menck HR Two hundred eighty-six cases of parathyroid carcinoma treated in the U.S. between 1985-1995: a National Cancer Data Base Report. The American College of Surgeons Commission on Cancer and the American Cancer Society Cancer 1999 86 538 544 10.1002/(SICI)1097-0142(19990801)86:3<538::AID-CNCR25>3.0.CO;2-K 10430265

[B10] DeLellis RA Parathyroid carcinoma: an overview Adv Anat Pathol 2005 12 53 61 10.1097/01.pap.0000151319.42376.d4 15731573

[B11] Westin G Björklund P Åkerström G Molecular genetics of parathyroid disease World J Surg 2009 33 2224 2233 10.1007/s00268-009-0022-6 19373510

[B12] Haven CJ van Puijenbroek M Tan MH Teh BT Fleuren GJ van Wezel T Morreau H Identification of MEN1 and HRPT2 somatic mutations in paraffin-embedded (sporadic) parathyroid carcinomas Clin Endocrinol 2007 67 370 376 10.1111/j.1365-2265.2007.02894.x 17555500

[B13] Vasef MA Brynes RK Sturm M Bromley C Robinson RA Expression of cyclin D1 in parathyroid carcinomas, adenomas, and hyperplasias: a paraffin immunohistochemical study Mod Pathol 1999 12 412 416 10229506

[B14] Carpten JD Robbins CM Villablanca A Forsberg L Presciuttini S Bailey-Wilson J Simonds WF Gillanders EM Kennedy AM Chen JD Agarwal SK Sood R Jones MP Moses TY Haven C Petillo D Leotlela PD Harding B Cameron D Pannett AA Höög A Heath H James-Newton LA Robinson B Zarbo RJ Cavaco BM Wassif W Perrier ND Rosen IB Kristoffersson U Turnpenny PD Farnebo LO Besser GM Jackson CE Morreau H Trent JM Thakker RV Marx SJ Teh BT Larsson C Hobbs MR HRPT2, encoding parafibromin, is mutated in hyperparathyroidism-jaw tumor syndrome Nat Genet 2002 32 676 680 10.1038/ng1048 12434154

[B15] Okamoto T Iihara M Obara T Tsukada T Parathyroid carcinoma: etiology, diagnosis, and treatment World J Surg 2009 33 2343 2354 10.1007/s00268-009-9999-0 19350316

[B16] Tan MH Morrison C Wang P Yang X Haven CJ Zhang C Zhao P Tretiakova MS Korpi-Hyovalti E Burgess JR Soo KC Cheah WK Cao B Resau J Morreau H The BT Loss of parafibromin immunoreactivity is a distinguishing feature of parathyroid carcinoma Clin Cancer Res 2004 10 6629 6637 10.1158/1078-0432.CCR-04-0493 15475453

[B17] Juhlin C Larsson C Yakoleva T Leibiger I Leibiger B Alimov A Weber G Höög A Villablanca A Loss of parafibromin expression in a subset of parathyroid adenomas Endocr Relat Cancer 2006 13 509 523 10.1677/erc.1.01058 16728578

[B18] Juhlin CC Villablanca A Sandelin K Haglund F Nordenström J Forsberg L Bränström R Obara T Arnold A Larsson C Höög A Parafibromin immunoreactivity: its use as an additional diagnostic marker for parathyroid tumor classification Endocr Relat Cancer 2007 14 501 512 10.1677/ERC-07-0021 17639063

[B19] Howell VM Gill A Clarkson A Nelson AE Dunne R Delbridge LW Robinson BG Teh BT Gimm O Marsh DJ Accuracy of combined protein gene product 9.5 and parafibromin markers for immunohistochemical diagnosis of parathyroid carcinoma J Clin Endocrinol Metab 2009 94 434 441 10.1210/jc.2008-1740 19017757

[B20] Juhlin CC Haglund F Villablanca A Forsberg L Sandelin K Bränström R Larsson C Höög A Loss of expression for the Wnt pathway components adenomatous polyposis coli and glycogen synthase kinase 3-beta in parathyroid carcinomas Int J Oncol 2009 34 481 492 19148484

[B21] Giles RH van Es JH Clevers H Caught up in a Wnt storm: Wnt signaling in cancer Biochim Biophys Acta 2003 1653 1 24 12781368 10.1016/s0304-419x(03)00005-2

[B22] Lustig B Behrens J The Wnt signaling pathway and its role in tumor development J Cancer Res Clin Oncol 2003 129 199 221 12707770 10.1007/s00432-003-0431-0 PMC12161963

[B23] Klaus A Birchmeier W Wnt signalling and its impact on development and cancer Nat Rev Cancer 2008 8 387 398 10.1038/nrc2389 18432252

[B24] MacDonald BT Tamai K He X Wnt/beta-catenin signaling: components, mechanisms, and diseases Dev Cell 2009 17 9 26 10.1016/j.devcel.2009.06.016 19619488 PMC2861485

[B25] Esteller M Sparks A Toyota M Sanchez-Cespedes M Capella G Peinado MA Gonzalez S Tarafa G Sidransky D Meltzer SJ Baylin SB Herman JG Analysis of adenomatous polyposis coli promoter hypermethylation in human cancer Cancer Res 2000 60 4366 4371 10969779

[B26] Virmani AK Rathi A Sathyanarayana UG Padar A Huang CX Cunnigham HT Farinas AJ Milchgrub S Euhus DM Gilcrease M Herman J Minna JD Gazdar AF Aberrant methylation of the adenomatous polyposis coli (APC) gene promoter 1A in breast and lung carcinomas Clin Cancer Res 2001 7 1998 2004 11448917

[B27] Segditsas S Sieber OM Rowan A Setien F Neale K Phillips RK Ward R Esteller M Tomlinson IP Promoter hypermethylation leads to decreased APC mRNA expression in familial polyposis and sporadic colorectal tumours, but does not substitute for truncating mutations Exp Mol Pathol 2008 85 201 206 10.1016/j.yexmp.2008.09.006 18977219

[B28] Fu X Li J Li K Tian X Zhang Y Hypermethylation of APC promoter 1A is associated with moderate activation of Wnt signalling pathway in a subset of colorectal serrated adenomas Histopathology 2009 55 554 563 10.1111/j.1365-2559.2009.03411.x 19912361

[B29] Ignatov A Bischoff J Ignatov T Schwarzenau C Krebs T Kuester D Costa SD Roessner A Semczuk A Schneider-Stock R APC promoter hypermethylation is an early event in endometrial tumorigenesis Cancer Sci 2010 101 321 327 10.1111/j.1349-7006.2009.01397.x 19900189 PMC11158934

[B30] Plimack ER Kantarjian HM Issa JP Decitabine and its role in the treatment of hematopoietic malignancies Leuk Lymphoma 2007 48 1472 1481 10.1080/10428190701471981 17701577

[B31] Daskalakis M Blagitko-Dorfs N Hackanson B Decitabine Recent Results Cancer Res 2010 184 131 157 full_text 20072836 10.1007/978-3-642-01222-8_10

[B32] Segersten U Correa P Hewison M Hellman P Dralle H Carling T Åkerström G Westin G 25-hydroxyvitamin D(3)-1alpha-hydroxylase expression in normal and pathological parathyroid glands J Clin Endocrinol Metab 2002 87 2967 2972 10.1210/jc.87.6.2967 12050281

[B33] Björklund P Åkerström G Westin G Accumulation of nonphosphorylated ß- catenin and c-myc in primary and uremic secondary hyperparathyroid tumors J Clin Endocrinol Metab 2007 92 338 344 10.1210/jc.2006-1197 17047023

[B34] Wei SC Su YN Tsai-Wu JJ Wu CH Huang YL Sheu JC Wang CY Wong JM Genetic analysis of the APC gene in Taiwanese familial adenomatous polyposis J Biomed Sci 2004 11 260 265 10.1007/BF02256569 14966376

[B35] Ikediobi ON Davies H Bignell G Edkins S Stevens C O'Meara S Santarius T Avis T Barthorpe S Brackenbury L Buck G Butler A Clements J Cole J Dicks E Forbes S Gray K Halliday K Harrison R Hills K Hinton J Hunter C Jenkinson A Jones D Kosmidou V Lugg R Menzies A Mironenko T Parker A Perry J Raine K Richardson D Shepherd R Small A Smith R Solomon H Stephens P Teague J Tofts C Varian J Webb T West S Widaa S Yates A Reinhold W Weinstein JN Stratton MR Futreal PA Wooster R Mutation analysis of 24 known cancer genes in the NCI-60 cell line set Mol Cancer Ther 2006 5 2606 2612 10.1158/1535-7163.MCT-06-0433 17088437 PMC2705832

[B36] Ronaghi M Uhlén M Nyrén P A sequencing method based on real-time pyrophosphate Science 1998 281 363 365 10.1126/science.281.5375.363 9705713

[B37] Colella S Shen L Baggerly KA Issa JP Krahe R Sensitive and quantitative universal Pyrosequencing methylation analysis of CpG sites Biotechniques 2003 35 146 150 12866414 10.2144/03351md01

[B38] Tost J Dunker J Gut IG Analysis and quantification of multiple methylation variable positions in CpG islands by Pyrosequencing Biotechniques 2003 35 152 156 12866415 10.2144/03351md02

[B39] van Noort M Meeldijk J van der Zee R Destree O Clevers H Wnt signaling controls the phosphorylation status of beta-catenin J Biol Chem 2002 277 17901 17905 10.1074/jbc.M111635200 11834740

[B40] Segersten U Björklund P Hellman P Åkerström G Westin G Potentiating effects of nonactive/active vitamin D analogues and ketoconazole in parathyroid cells Clin Endocrinol (Oxf) 2007 66 399 404 10.1111/j.1365-2265.2006.02746.x 17302875

[B41] Bender CM Pao MM Jones PA Inhibition of DNA methylation by 5-aza-2'- deoxycytidine suppresses the growth of human tumor cell lines Cancer Res 1998 58 95 101 9426064

[B42] Jin B Yao B Li JL Fields CR Delmas AL Liu C Robertson KD DNMT1 and DNMT3B modulate distinct polycomb-mediated histone modifications in colon cancer Cancer Res 2009 69 7412 7421 10.1158/0008-5472.CAN-09-0116 19723660 PMC2745494

[B43] Busaidy NL Jimenez C Habra MA Schultz PN El-Naggar AK Clayman GL Asper JA Diaz EM Jr Evans DB Gagel RF Garden A Hoff AO Lee JE Morrison WH Rosenthal DI Sherman SI Sturgis EM Waguespack SG Weber RS Wirfel K Vassilopoulou-Sellin R Parathyroid carcinoma: a 22-year experience Head Neck 2004 26 716 726 10.1002/hed.20049 15287039

[B44] Laird PW Jackson-Grusby L Fazeli A Dickinson SL Jung WE Li E Weinberg RA Jaenisch R Suppression of intestinal neoplasia by DNA hypomethylation Cell 1995 81 197 205 10.1016/0092-8674(95)90329-1 7537636

[B45] Cantor JP Iliopoulos D Rao AS Druck T Semba S Han SY McCorkell KA Lakshman TV Collins JE Wachsberger P Friedberg JS Huebner K Epigenetic modulation of endogenous tumor suppressor expression in lung cancer xenografts suppresses tumorigenicity Int J Cancer 2007 120 24 31 10.1002/ijc.22073 17019711

[B46] Venturelli S Armeanu S Pathil A Hsieh CJ Weiss TS Vonthein R Wehrmann M Gregor M Lauer UM Bitzer M Epigenetic combination therapy as a tumor selective treatment approach for hepatocellular carcinoma Cancer 2007 109 2132 2141 10.1002/cncr.22652 17407132

[B47] Issa JP Kantarjian HM Targeting DNA methylation Clin Cancer Res 2009 15 3938 3946 10.1158/1078-0432.CCR-08-2783 19509174 PMC2732562

