# Supplementary material for: Aberrant WNT/β-catenin signaling in parathyroid carcinoma
Source: Mol Cancer. 2010 Nov 15;9:294. doi: 10.1186/1476-4598-9-294 (PMC2993678; doi:10.1186/1476-4598-9-294)
Supplement: Additional file 1 — Table S1 - Quantitative bisulfite pyrosequencing of 10 CpGs in the APC promoter 1A. Normal parathyroid tissue specimens (PN, n = 8) and parathyroid carcinoma (PC, n = 5) were analyzed in triplicate. [file 1476-4598-9-294-S1.DOC]

| **Tissue sample** | **Mean CpG 1 (%)** | **Mean CpG 2 (%)** | **Mean CpG 3 (%)** | **Mean CpG 4 (%)** | **Mean CpG 5 (%)** | **Mean CpG 6 (%)** | **Mean CpG 7 (%)** | **Mean CpG 8 (%)** | **Mean CpG 9 (%)** | **Mean CpG 10 (%)** | **Mean all CpGs (%)** |
| --- | --- | --- | --- | --- | --- | --- | --- | --- | --- | --- | --- |
| **PN1** | 19±2.6 | 17±3.6 | 19.3±2.5 | 23±3.5 | 7.3±2.1 | 20±2.0 | 11.3±1.2 | 13±2.6 | 10.3±1.2 | 18.7±3.1 | 15.9±5.3 |
| **PN3** | 21.3±3.2 | 21±1.7 | 20.3±1.5 | 21.3±1.5 | 8.7±1.2 | 19.7±2.9 | 12.3±1.5 | 14.3±1.5 | 12.3±1.2 | 20±1.7 | 17.1±4.8 |
| **PN4** | 23.3±1.5 | 23±1.0 | 19.7±2.1 | 22.3±1.2 | 7.3±0.6 | 22.3±1.2 | 10.7±1.2 | 16±1.0 | 13.3±0.6 | 20.3±1.2 | 17.8±5.6 |
| **PN5** | 23.3±3.1 | 23.3±2.5 | 21.7±2.9 | 22.7±3.2 | 7.3±0.6 | 21±2.6 | 12±1.7 | 16.7±1.5 | 12.7±1.2 | 21.3±0.6 | 18.2±5.8 |
| **PN6** | 19.7±2.1 | 18.3±2.5 | 18.7±4.5 | 21.7±2.5 | 7±1.7 | 20±1.7 | 11±1.0 | 14.7±1.5 | 12.3±2.1 | 19.7±2.3 | 16.3±5.0 |
| **PN7** | 16.3±2.5 | 15±1.7 | 18.7±5.5 | 23.3±2.1 | 7±1.0 | 18±2.0 | 9.3±1.5 | 11.7±1.2 | 10.3±0.6 | 17.3±0.6 | 14.7±5.2 |
| **PN8** | 8.3±1.5 | 6.3±1.5 | 19±7.9 | 17.3±4.0 | 5±1.0 | 11±2.6 | 6.3±1.5 | 6.3±1.5 | 5.7±1.5 | 9.7±2.1 | 9.5±5.5 |
| **PN9** | 16±1.7 | 15.7±2.3 | 18±1.7 | 15.7±0.6 | 6±0.0 | 15±1.7 | 9.3±1.2 | 12.3±1.2 | 9.7±0.6 | 14.7±2.3 | 13.2±3.9 |
| **PC1** | 69.7±3.8 | 51±5.3 | 43±5.2 | 47±4.6 | 41±1.0 | 50.3±4.2 | 42±1.0 | 40±4.4 | 39.3±2.3 | 57.7±5.5 | 48.1±9.8 |
| **PC2** | 76±3.0 | 76.3±0.6 | 67.7±4.2 | 73±1.7 | 64±1.0 | 81.3±3.8 | 65.7±1.5 | 65.3±1.2 | 64.7±1.5 | 84±4.6 | 71.8±7.4 |
| **PC3** | 51±3.6 | 49.7±3.1 | 44.7±6.0 | 49.7±2.5 | 47.3±2.5 | 54.3±2.9 | 47.7±2.5 | 43.7±3.1 | 44.3±4.0 | 56.7±4.9 | 48.9±5.1 |
| **PC4** | 92.3±0.6 | 89.7±0.6 | 85.3±2.5 | 89.3±0.6 | 82.7±0.6 | 98.7±1.2 | 86.3±1.2 | 81.7±1.2 | 80.7±1.2 | 93.7±7.8 | 88.0±6.0 |
| **PC5** | 52±3.0 | 50.7±2.5 | 44.7±4.2 | 52.3±2.1 | 48±2.0 | 55.3±2.9 | 47.3±2.9 | 44±2.0 | 44.7±1.5 | 59±5.2 | 49.8±5.4 |

Table S1. Quantitative bisulfite pyrosequencing of 10 CpGs in the APC promoter 1A. Normal parathyroid tissue specimens (PN, n=8) and parathyroid carcinoma (PC, n=5) were analyzed in triplicate.
